# Selection of variables for multivariable models: Opportunities and limitations in quantifying model stability by resampling

**DOI:** 10.1002/sim.8779

**Published:** 2020-10-21

**Authors:** Christine Wallisch, Daniela Dunkler, Geraldine Rauch, Riccardo de Bin, Georg Heinze

**Affiliations:** ^1^ Center for Medical Statistics, Informatics and Intelligent Systems, Section for Clinical Biometrics Medical University of Vienna Vienna Austria; ^2^ Institute of Biometry and Clinical Epidemiology Charité ‐ Universitätsmedizin Berlin, Corporate Member of Freie Universität Berlin, Humboldt‐Universität zu Berlin, and Berlin Institute of Health Berlin Germany; ^3^ Berlin Institute of Health Berlin Germany; ^4^ Department of Mathematics University of Oslo Oslo Norway

**Keywords:** backward elimination, bootstrap, stability measures, subsampling, variable selection

## Abstract

Statistical models are often fitted to obtain a concise description of the association of an outcome variable with some covariates. Even if background knowledge is available to guide preselection of covariates, stepwise variable selection is commonly applied to remove irrelevant ones. This practice may introduce additional variability and selection is rarely certain. However, these issues are often ignored and model stability is not questioned.

Several resampling‐based measures were proposed to describe model stability, including variable inclusion frequencies (VIFs), model selection frequencies, relative conditional bias (RCB), and root mean squared difference ratio (RMSDR). The latter two were recently proposed to assess bias and variance inflation induced by variable selection. Here, we study the consistency and accuracy of resampling estimates of these measures and the optimal choice of the resampling technique. In particular, we compare subsampling and bootstrapping for assessing stability of linear, logistic, and Cox models obtained by backward elimination in a simulation study. Moreover, we exemplify the estimation and interpretation of all suggested measures in a study on cardiovascular risk. The VIF and the model selection frequency are only consistently estimated in the subsampling approach. By contrast, the bootstrap is advantageous in terms of bias and precision for estimating the RCB as well as the RMSDR. Though, unbiased estimation of the latter quantity requires independence of covariates, which is rarely encountered in practice. Our study stresses the importance of addressing model stability after variable selection and shows how to cope with it.

## 1 INTRODUCTION

Statistical models are often used in medical research to describe the association of an outcome of interest with several explanatory variables by means of a simple mathematical rule. In some applications of statistical models, one may be interested in predicting the outcome variable with the explanatory variables, for example, when predicting a person's cardiovascular risk at a health screening. In other applications, one may want to quantify the effects of prognostic factors on the expected value of an outcome variable, for example, when assessing the predictive value of a cardiovascular risk factor or when estimating its causal effect on the outcome variable.^1^ (In the remainder, we will use the more neutral term “covariate” exchangeably for explanatory variable, independent variable, risk factor, prognostic factor, and so on.) In any case, at the beginning of the analysis many covariates may be available for inclusion in a model, but it is not always clear upfront if all or just some of them should be included in the final model. Even with a fair amount of domain knowledge to support the modeling process, researchers often apply data‐driven selection procedures to determine the set of covariates for their model. Statistical software packages offering such algorithms, for example, backward elimination (BE) of variables based on iteratively assessing their “significance,” report the final model with the estimates of the regression coefficients and their SEs, as if this model had been prespecified. Consequently, it has become common practice in the medical literature to present models resulting from selection procedures without any adjustment or even without any note of caution in their interpretation. However, it has been known for a long time that models derived by selection procedures are instable and even small modifications of the data may lead to important changes in the set of included covariates, and consequently in the magnitude of the estimated regression coefficients.^2^ In fact, a possibly large amount of uncertainty is too often simply ignored.

As we explained in our recent review article on variable selection, wrong inclusion or exclusion of covariates may become manifest in bias and (increased or decreased) variance of regression coefficients.^3^ However, bias and variance due to selection may be left unnoticed as they cannot easily be assessed in real data analyses. Therefore, in addition to reporting how “certain” the selection of each covariate is, it is desirable to be able to quantify the effect of applying a variable selection algorithm on bias and variance of regression coefficients. With appropriate measures routinely reported by software packages, decisions in favor of or against variable selection procedures would have an empirical basis. In addition to the previously proposed variable inclusion frequency (VIF) and model selection frequency (MSF),^4^, ^5^, ^6^ we suggested to quantify the effect of selection on bias by estimation of the relative conditional bias (RCB) and to quantify the additional variability by the root mean squared difference ratio (RMSDR).^3^ These quantities are usually computed by resampling. As a resampling scheme for VIF and MSF, the nonparametric bootstrap, that is, sampling with replacement, is commonly applied.^7^ However, as outlined in several recent articles,^8^, ^9^, ^10^ subsampling, that is, sampling without replacement, may often be more attractive. So far, no comprehensive simulation study has compared results from bootstrapping or subsampling VIFs or MSFs with their estimands. By estimands we mean the quantities that the sample estimator of the statistic should estimate given a well‐defined population from which a random sample of a particular size is available for estimation. (While a population quantity is a feature of the underlying population, the estimand is a feature of a statistical procedure applied to a finite sample of the population.) It is not known whether subsampling or bootstrapping is preferable to estimate RCB and RMSDR, and how well estimates of RCB and RMSDR compare with their estimands.

The first objective of this article is to provide definitions of the estimands for VIF, MSF, RCB, and RMSDR. The second objective is to evaluate by simulation whether bootstrapping or subsampling is preferable to estimate the four measures in finite samples when different selection procedures are applied, and to study the large sample behavior of our estimands of interest and their estimators. As a third objective, we show the use of VIF, MSF, RCB, and RMSDR in a real example. We will focus on BE with the Akaike information criterion (AIC) as a stopping criterion, and report results for BE with *α* = .05 and penalized likelihood estimation with the least angle shrinkage and selection operator (Lasso, using 10‐fold cross‐validation to select the strength of the penalty) in the Supplementary Material. Our methodology is applicable with any other selection algorithm. We will concentrate on linear predictor models, in particular on linear regression, logistic regression and Cox regression, as these are the most popular statistical models applied in medical research.

The remainder of the article is organized as follows. Section 2 will define the estimands and sample estimators for VIF, MSF, RCB, and RMSDR. Subsequently, Section 3 will describe aims, methodology and results of a comprehensive simulation study on the four stability measures. In Section 4, a real life example will exemplify the use of these measures to quantify the instability of data‐driven variable selection. The article will conclude with a discussion and some practical recommendations.

## 2 PREREQUISITES

### 2.1 Linear predictor models

Continuous, binary and time‐to‐event outcomes are often analyzed with linear, logistic, or Cox regression models, respectively. The linear model is given by *Y* = *β*_0_ + *β*_1_*X*_1_ + … + *β*_*k*_*X*_*k*_ + *ϵ*, where *Y* is a continuous outcome, *X*_1_, …, *X*_*k*_ are covariates, *β*_0_, *β*_1_, …, *β*_*k*_ are the regression coefficients, and *ϵ* is normally distributed with unknown variance *σ*^2^. In the logistic model, the probability that the binary outcome variable *Y* assumes the value 1 is modeled as *Pr*(*Y* = 1) = *π* = *expit*(*β*_0_ + *β*_1_*X*_1_ + … + *β*_*k*_*X*_*k*_), where expit(*z*) = exp(*z*)/[1 + exp(*z*)]. The Cox proportional hazards model estimates the hazard function *h*(*t*) = *f*(*t*)/*S*(*t*), that is, the density divided by the survivor function. Here, *t* denotes time, and the hazard is modeled as a function of time and covariates as *h*(*X*, *t*) = *h*_0_(*t*)exp(*β*_1_*X*_1_ + … + *β*_*k*_*X*_*k*_), where *h*_0_(*t*) is an unspecified baseline hazard function. Modelling the hazard is an elegant way to circumvent the problem of only partial observability of the outcome, the time to the event of interest *T*. In fact, if *C* denotes the random variable of follow‐up times, only *Y* = min(*T*, *C*) is observable.

The three models have in common that they use a linear predictor, *β*_1_*X*_1_ + … + *β*_*k*_*X*_*k*_, and hence the interpretation of the regression coefficients *β*_*j*_; *j* = 1, …, *k*; is straightforward as the expected difference in outcome (or log odds or log hazard) when comparing two subjects who differ in *X*_*j*_ by 1 unit and who have equal values in all other variables *X*_*l*_ ≠ *X*_*j*_. In all three models, regression coefficients are estimated by maximizing the (partial) likelihood of the model given the observed data *y*, $L\left(\beta y\right)=\prod_{i=1}^{N}f_{i}\left(y_{i}\beta x_{i}\right)$. Here, we assume that a sample of size *N* of the outcome variable and the covariates is observed, that *y* denotes the vector of observed values *y*_*i*_, *i* = 1, …, *N*, of the outcome variable, and that *x*_*i*_ is the covariate row vector for subject *i*. In linear regression, maximum likelihood estimation is equivalent to minimizing the sum of the observed squared residuals. A *P*‐value for testing the null hypothesis that *β*_*j*_ = 0 can be obtained either by the Wald procedure, assuming that the test statistic $z_{j}={\hat{\beta }}_{j}/{\hat{\sigma }}_{j}$ follows a standard normal distribution under the null hypothesis (a *t*‐distribution is used for linear regression), or by employing likelihood theory, claiming that the likelihood ratio statistic follows a *χ*^2^‐distribution.

### 2.2 Preselection of candidate covariates

Statistical methods cannot distinguish between spurious and real associations between variables. Therefore, it is important to predefine a set of candidate covariates for which domain expertise would assume or at least hypothesize some association with the dependent variable.^3^ In the sequel we will denote the model consisting of such covariates as the global model. Model building could be concluded by estimating that global model. However, in many applications researchers may wish to intentionally deviate from the assumed “ground truth” of the global model and report a descriptive model that captures the main associations only and omits the negligible ones. This motivates the application of statistical variable selection algorithms.

### 2.3 Selection algorithm

Given a particular significance level *α*_*B*_, BE starts with fitting the global model including all preselected candidate covariates, and then iteratively eliminates the least significant covariate and refits the model until all *P*‐values are lower than the prespecified *α*_*B*_. Selecting *α*_*B*_ = 0.157 is often a sensible choice and approximately equivalent to eliminating covariates until the AIC can no longer be improved.^3^, ^11^, ^12^ We will abbreviate BE with *α*_*B*_ = 0.157 by BE(AIC).

BE can be seen as a selection procedure which forces some regression coefficient estimates to be exactly 0. In the following, we will denote by $\tilde{\beta }$ and ${\tilde{\sigma }}^{2}$ the estimates of the regression coefficients and their variances from the global model including all covariates (*X*_1_, …, *X*_*k*_), respectively, and by $\hat{\beta }$ the estimates resulting from applying BE(AIC). We define by *J* the set of indices of covariates with true *β*_*j*_ ≠ 0 (the predictors, *j* ∈ *J*). Correspondingly, *J*^′^ denotes the set of indices of covariates with no effect (the nonpredictors, $\beta_{j^{\prime }}=0,j^{\prime }\in J^{\prime }$).

### 2.4 Resampling schemes

Bootstrap resampling consists of drawing *B* resamples of the original dataset, each containing *N* observations. This procedure is also called the nonparametric bootstrap and requires that observations are independently and identically distributed.^13^, ^14^ By using sampling with replacement, some observations may appear multiple times in a single resample, while others may be missing. By contrast, subsampling consists of drawing *B* subsets of *m* < *N* observations from the original dataset. Often, *m* = 0.632*N* is chosen as 0.632 is the probability with which an observation appears in a bootstrap sample. For comparability with the bootstrap, Sauerbrei et al used S_0.632_ in an investigation of model stability.^15^ However, there is no general agreement on the choice of *m*. Here, we consider *m* = 0.5*N* (S_0.5_), *m* = ⌊0.632*N*⌋ (S_0.632_), and *m* = 0.8*N* (S_0.8_). Moreover, it was noted that with high‐dimensional regularized Cox regression, sampling without replacement may lead to more accurate prediction error estimates than sampling with replacement.^16^ The sampling distribution of a regression coefficient can be equivalently approximated by the bootstrap or subsampling when appropriately weighting the estimator (Supplementary Material S1).^17^, ^18^

## 3 STABILITY MEASURES

### 3.1 Estimands

The definition of estimands of the four stability measures as expected values can be found in Table 1. A large number *Q* of datasets are generated with a given data generating mechanism for covariates and the outcome variable, for example, based on a regression model with fixed regression coefficients *β* (see section 4.1 for details on the data generating mechanism in this simulation study). With each simulated dataset (*q* = 1, …, *Q*), we estimate ${\hat{\beta }}^{q}$ and subsequently, approximate the estimands according to the formulas in Table 1. The expected values can be conveniently approximated with an arbitrary small error by adjusting *Q*.

**TABLE 1:** Estimands describing the uncertainty of model estimation incurred by variable selection, their approximation by simulation, and their estimation by resampling

Estimand	Definition	Approximationby simulation	Resampling‐based estimator
VIF_*j*_	$E\left[I\left({\hat{\beta }}_{j}\neq 0\right)\right]$	$\sum_{q=1}^{Q}I\left({\hat{\beta }}_{j}^{q}\neq 0\right)/Q$	$\sum_{b=1}^{B}I\left({\hat{\beta }}_{j}^{b}\neq 0\right)/B$
MSF(*J*)	$E\left[\prod_{j\in J}I\left({\hat{\beta }}_{j}\neq 0\right)\cdot \prod_{j{}^{\prime }\in J{}^{\prime }}I\left({\hat{\beta }}_{j{}^{\prime }}=0\right)\right]$	$\sum_{q=1}^{Q}\prod_{j\in J}I\left({\hat{\beta }}_{j}^{q}\neq 0\right)\cdot \prod_{j{}^{\prime }\in J{}^{\prime }}I\left({\hat{\beta }}_{j^{\prime }}^{q}=0\right)/Q$	$\sum_{b=1}^{B}\prod_{j\in J}I\left({\hat{\beta }}_{j}^{b}\neq 0\right)\cdot \prod_{j^{\prime }\in J{}^{\prime }}I\left({\hat{\beta }}_{j^{\prime }}^{b}=0\right)/B$
RCB_*j*_	$\left(\frac{E\left({\hat{\beta }}_{j}\right)}{\beta_{j}\cdot \mathrm{VI}F_{j}}-1\right)$	$\left(\frac{\sum_{q=1}^{Q}{\hat{\beta }}_{j}^{q}}{\beta_{j}\cdot \mathrm{VI}F_{j}\cdot Q}-1\right)$	$\left(\frac{\sum_{b=1}^{B}{\hat{\beta }}_{j}^{b}}{{\tilde{\beta }}_{j}\cdot \hat{\mathrm{VI}F_{j}}\cdot B}-1\right)$
RMSDR_*j*_	$\sqrt{\frac{E{\left({\hat{\beta }}_{j}-\beta_{j}\right)}^{2}}{E{\left({\tilde{\beta }}_{j}-\beta_{j}\right)}^{2}}}$	$\sqrt{\frac{\sum_{q=1}^{Q}{\left({\hat{\beta }}_{j}^{q}-\beta_{j}\right)}^{2}/Q}{\sum_{q=1}^{Q}{\left({\tilde{\beta }}_{j}^{q}-\beta_{j}\right)}^{2}/Q}}$	$\sqrt{\frac{\sum_{b=1}^{B}{\left({\hat{\beta }}_{j}^{b}-{\tilde{\beta }}_{j}\right)}^{2}/B}{{\tilde{\sigma }}_{j}^{2}}}$

*Note:* Superscripts *q* or *b* indicate estimates obtained in the *q*th simulated dataset or the *b*th resample, respectively. Estimates ${\hat{\beta }}_{j}$ and ${\hat{\beta }}_{j}^{b}$are set to 0 if they are not selected by the variable selection algorithm in the corresponding model. For the sets *J* and *J*′ of indices, *J* ∪ *J*^′^ = {1, …, *k*}, and *J* ∩ *J*^′^ = { }. *I*(·) is the indicator function, that is, it is 1 if the expression (·) is true and 0 otherwise.

Abbreviations: MSF, model selection frequency; RCB, relative conditional bias; RMSDR, root mean squared difference ratio; VIF, variable inclusion frequency.

In particular, the VIF of a covariate *X*_*j*_, denoted by VIF_*j*_, describes how likely the covariate is selected. It depends, among other things, on the selection method (eg, BE(AIC)) and on *N* as the selection is strongly determined by the power of the Wald/likelihood‐ratio test.

The MSF(*J*) describes how likely the true model was selected, which exactly includes the covariates *X*_*j*_, *j* ∈ *J*. The MSF depends on the number of covariates *k*, and the number of possible models, 2^*k*^, sharply increases with increasing *k*. Thus, a small MSF must be expected even with reliable variable selection algorithms and moderate values of *k*.

The RCB_*j*_ of a regression coefficient *β*_*j*_ measures the bias of an estimate obtained after a selection procedure with respect to the true value. It can be conveniently expressed as a percentage, where values greater than 0 mean stronger overestimation and values less than 0 underestimation. The true RCB is only defined for covariates with a nonzero regression coefficient (predictors) and it can only be estimated if the corresponding covariate has been selected by the selection procedure.

RCB is related to the idea of parameterwise shrinkage factors (PSFs) proposed by Sauerbrei to correct for overestimation of regression coefficients in models selected via selection procedures.^19^ Assuming that a regression coefficient is overestimated, which leads to an RCB > 0, the idea is that the overestimation can be corrected by shrinking the regression coefficient by a suitable factor. Instead of PSFs, we can use (1 + RCB)^−1^. Such a shrinkage factor (SF) could be used to correct bias, as estimated by resampling, when applying variable selection. By contrast, PSFs ignore alternative models obtained by variable selection and correct the bias relative to a “least false model,” which is given by the expected values of the regression coefficients if the selected model was the true model.

The RMSDR_*j*_ measures the inflation in root mean squared error (RMSE) relative to the SE of the estimate from the global model including all covariates. Values >1 indicate inflation of mean squared error caused by applying a variable selection procedure, while values <1 indicate a reduction.

### 3.2 Estimators

The definitions of all estimands require knowledge of the true model and therefore, estimates cannot easily be obtained in the analysis of a real dataset. As demonstrated in Table 1, the estimators of the four stability measures can be defined by plugging‐in the unbiased estimates $\tilde{\beta }$ and ${\tilde{\sigma }}^{2}$ from the global model for their true quantities and by using *B* resamples to approximate the sampling distribution of $\hat{\beta }$. The definitions in Table 1 apply to any selection or resampling procedure.

## 4 SIMULATION STUDY

### 4.1 Design

In this comprehensive simulation study, we applied BE(AIC) in linear regression models to evaluate the proposed stability measures. We also applied BE with *α*_*B*_ = 0.05 (BE(0.05)) and the Lasso,^20^ and extended the simulation to logistic and Cox regression (details on the design can be found in Supplementary Material S8‐S9). We use the structured approach ADEMP^21^ to describe our simulation study.

**Aims:** The aims of the study were (1) to assess consistency of the proposed stability measures and (2) to evaluate how accurately they can be estimated in small to large datasets.

**Data‐generating mechanism:** Nine continuous and six categorical covariates were generated in order to obtain data mimicking a typical medical observational study.^22^ While the key aspects of the data generation are described here, we refer to the technical report accompanying Binder et al^22^ for a comprehensive description.

First, normal deviates *Z*_*l*_, *l* = 1, …, 15 were generated from a multivariate standard Gaussian distribution with prespecified correlations ranging from −0.3 to 0.8. Subsequently, the normal deviates were transformed to yield plausible marginal distributions of 15 “real” variables, including two ordinal factors with three levels each.^22^ Thus, 17 design variables *X*_*j*_, *j* = 1, …, 17, resulted, and we assumed that only *X*_1_, …, *X*_8_ had a nonzero effect on the outcome (see Table 2 for the regression coefficients, and Supplementary Material S2 for distributions and correlation structure). Hence, *J* = {1, …, 8} and *J*^′^ = {9, …, 17}. A continuous outcome variable *Y* was generated from $Y∼N\left(\sum_{j=1}^{8}x_{j}\beta_{j},0.932\right)$, resulting in an *R* ^2^ of 0.47.

**TABLE 2:** Setup of simulation study: Distribution, true regression coefficients, standardized regression coefficients, partial R,^2^ and multiple *R* ^2^ of variables

Variable	Distribution	True regression coefficient *β*_*j*_	Standardized regression coefficient *β*_*j*_*σ*_*j*_	Partial *R* ^2^ in linear regression (×100)	Multiple *R* ^2^/correlation (×100)
*X*_1_	normal	0.040	0.400	5.1	47.7
*X*_2_	normal	−1.040	−0.385	3.8	56.4
*X*_3_	zero‐inflated normal	0.250	0.394	3.4	66.4
*X*_4_	normal	0.624	0.249	2.3	40.3
*X*_5_	binomial(0.4)	0.402	0.194	2.0	12.6
*X*_6_	log‐normal	0.021	0.171	1.9	0
*X*_7_	binomial(0.7)	−0.398	−0.189	0.8	61.7
*X*_8_	normal	−0.009	−0.094	0.3	44.4
*X*_9_	binomial(0.7)	0	0	0	39.0
*X*_10_	binomial(0.2)	0	0	0	70.0
*X*_11_	exponential	0	0	0	15.6
*X*_12_	binomial(0.2)	0	0	0	34.5
*X*_13_	binomial(0.1)	0	0	0	34.0
*X*_14_	normal	0	0	0	36.4
*X*_15_	normal	0	0	0	0
*X*_16_	binomial(0.5)	0	0	0	17.7
*X*_17_	binomial(0.5)	0	0	0	0

We simulated *Q* = 1000 datasets with sample sizes 150, 300, 750, 1000, 5000, and 10 000. In each dataset we estimated the global model including all 17 design variables, and then applied BE(AIC).

**Estimands:** The estimands in this study were the stability measures VIF, MSF, RCB, and RMSDR, and their true values were approximated using the formulas provided in Table 1.

**Methods:** For each simulated dataset, we generated 1000 bootstrap (B) resamples and 1000 subsamples for each sampling proportion 0.5, 0.632, and 0.8 (denoted as S_0.5_, S_0.632_, and S_0.8_). BE(AIC) was applied to the resamples to estimate VIF, MSF, RCB, and RMSDR in each simulated dataset.

**Performance measures:** All resampling‐based estimates of stability measures were compared with their approximated estimands using mean estimates and RMSE for VIF and MSF, and median bias and median absolute bias for RCB and RMSDR. Expected Monte Carlo errors of all performance measures are acceptable and are reported in the Supplementary Material S3.

**Software:** R version 3.4.3 and packages *abe* 3.0.1, *data.table* 1.10.4‐3, *mvtnorm* 1.0‐7, *parallel* 3.4.3, were used.^23^

### 4.2 Simulation results

While the complete results of the simulation study are contained in the Supplementary Material S4 to S9, the typical performance of the estimators can already be understood by means of results selected for this article.

#### 4.2.1 Variable inclusion frequencies

**FIGURE 1:**
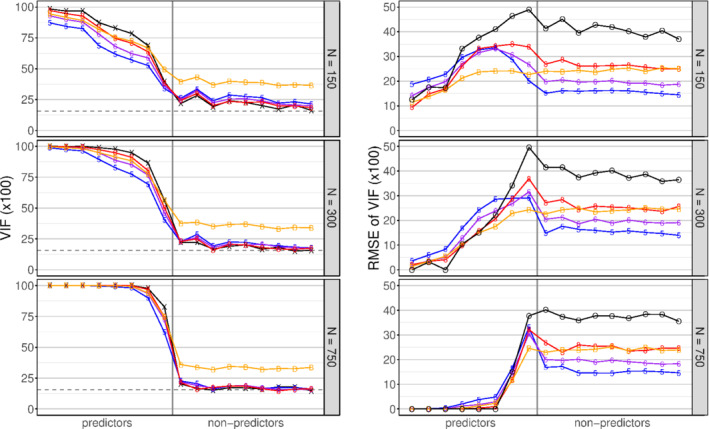
Simulation study: Variable inclusion frequencies (VIF) Left column: mean estimated VIF by subsampling with *m* = 0.5*N* (“5,” blue), *m* = 0.632*N* (“6,” purple), *m* = 0.8*N* (“8,” red), by bootstrap (“B,” yellow), and their estimands (“X,” black), for different sample sizes (top row, N = 150, middle N = 300, bottom N = 750). The horizontal dashed line represents the nominal significance level *α*_*B*_ = 0.157. Right column: root mean squared error (RMSE) of estimated VIF by subsampling with *m* = 0.5*N* (“5,” blue), *m* = 0.632*N* (“6,” purple), *m* = 0.8*N* (“8,” red), by bootstrap (“B,” yellow), and the omission/ selection strategy (“O,” black). The omission/selection strategy sets the VIF estimate to 0 or 1 according to omission or selection in the model fitted on a simulated dataset. Variables are ranked by partial R^2^ [Colour figure can be viewed at wileyonlinelibrary.com]

Approximated estimands of VIF, means, and RMSEs of estimated VIFs for all variables and three sample sizes (*N* ∈ {150, 300, 750}) are shown in Figure 1.

With a large sample size, estimands, and mean estimates of VIF for nonpredictors approached 0.157, in line with the nominal significance level corresponding to AIC selection, and were clearly higher for predictors. Subsampling estimated VIFs of nonpredictors almost unbiasedly, and among the subsampling proportions, S_0.5_ yielded the lowest RMSEs for nonpredictors. However, for predictors, B and S_0.8_ showed the smallest bias and B the lowest RMSEs. If VIFs were not estimated by resampling, but set to 0 or 1 depending on omission or selection of a covariate, then similarly low RMSEs could occasionally be reached for the strongest predictors, while the RMSEs for nonpredictors were clearly higher than with any resampling approach. Similar results were obtained for BE(0.05) and Lasso, as shown in the Supplementary Material S4.

#### 4.2.2 MSF of the true model

Model selection frequencies of the true model were most unbiasedly estimated by S_0.8_, followed by S_0.632_, S_0.5_ and B. For example, when N = 750, with S_0.8_ the mean frequency of selection of the correct model is 16.8%, very close to its estimand (18.8%). This value is fairly better than S_0.632_ and S_0.5_ (14.8% and 12.4%, respectively) and dramatically better than B (2.4%). However, the RMSE was smallest for S_0.5_ despite the underestimation, followed by S_0.632_, B and S_0.8_ (Table 3, Supplementary Material S5).

**TABLE 3:** Simulation study: Model selection frequency for the correct model

	N = 150		N = 300		N = 750	
Estimand	3.3		9.9		18.8	
Estimates	Mean	RMSE	Mean	RMSE	Mean	RMSE
S_0.5_	0.4	2.8	2.9	7.9	12.4	12.1
S_0.632_	1.0	3.2	5.1	8.4	14.8	13.9
S_0.8_	1.5	5.0	7.3	13.5	16.8	20.7
B	0.3	3.6	1.0	8.9	2.4	16.3

*Note:* Estimands, mean, and RMSE of estimates for subsampling with *m* = 0.5*N*, *m* = 0.632*N*, and *m* = 0.8*N* (S_0.5_, S_0.632_, S_0.8_) and bootstrap (B) estimators. All numbers multiplied by 100.

Abbreviation: RMSE, root mean squared error.

#### 4.2.3 Relative conditional bias

**FIGURE 2:**
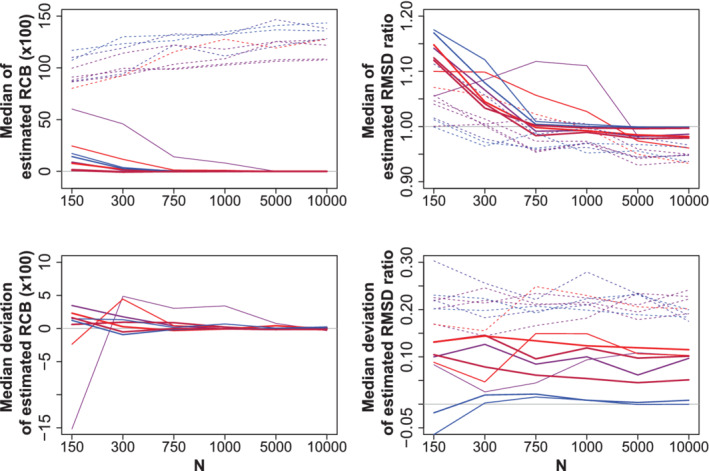
Simulation study: Relative conditional bias (left column) and root mean squared difference ratio (right column) estimated by bootstrap Upper row: median estimate; lower row: median deviation to the estimand; red and blue indicate high and low multiple correlation of a variable with others; solid and dashed lines represent predictors and nonpredictors. The line width is proportional to absolute effect size [Colour figure can be viewed at wileyonlinelibrary.com]

RCB could be estimated unbiasedly, at least with a large sample size, by all resampling approaches (Figure 2, left column and Supplementary Material S6). One predictor showed higher estimated RCBs, most likely because of its small effect size and the specific correlation structure. The median absolute deviation for B was less than for any subsampling method (Supplementary Material S6). For nonpredictors, the estimand is not defined but still estimates are obtained. They indicate that if a nonpredictor is selected, its regression coefficient is on average about twice as high as the coefficient in the global model.

#### 4.2.4 Root mean squared difference ratio

The estimand of RMSDR equals 1 if in its definition the selected model coefficient ${\hat{\beta }}_{j}$ is replaced by the global model coefficient ${\tilde{\beta }}_{j}$. The only difference in numerator and denominator of the RMSDR estimator in that case is that the numerator is obtained by resampling, while the denominator is the model‐based SE from the global model. The bootstrap estimator of the root mean squared deviation from ${\tilde{\beta }}_{j}$ converged to the model‐based SE much faster than any subsampling estimator (Supplementary Material S7). Therefore, we further considered bootstrap estimators of RMSDR only (Figure 2, right column).

Generally, the approximated estimands of RMSDR decreased with increasing sample size, reaching values below 0 at a sample size of 750 for most covariates, and at 5000 for all covariates. In this way, they indicate a benefit from applying variable selection with moderate or large sample sizes, but an increased error with smaller sample sizes. When investigating the median deviation of RMSDR estimates from their approximated estimands, a constant overestimation of the ratio by around 0.2 is observed for nonpredictors. For predictors, it is overestimated by approximately 0.1 if these predictors are correlated with others. RMSDR is almost unbiasedly estimated for uncorrelated predictors.

#### 4.2.5 Further results

With logistic and Cox regression, we observed approximately the same dependencies of approximated estimands on sample sizes and similar behavior of the estimators as in linear regression (Supplementary Material S8‐S9). The four stability measures described well the different operation characteristics of other variable selection procedures. For example, with BE(0.05) the RMSDRs at smaller sample size are greater than with BE(AIC), but with larger sample sizes they indicate more benefit, in particular for nonpredictors (Supplementary Material S7, Supplementary Figure 6B). This is because BE(0.05) removes nonpredictors more accurately than BE(AIC) with larger sample sizes. With the Lasso, RMSDR depends less on sample size than with BE(AIC). Because of the shrinkage of the Lasso estimator, RMSDR can be much smaller for nonpredictors and some predictors. However, because of the Lasso's characteristic to introduce bias towards zero, there are also predictors where the RMSDR is always greater than 1.

## 5 EXAMPLE

### 5.1 Aim

We illustrate the typical behavior of the estimators of VIF, MSF, RCB, and RMSDR by means of Cox regression analysis of a study of cardiovascular risk in healthy men. In our example, modeling was supposed to supply a concise description of the association of the hazard of cardiovascular events with covariates available at a preventive health screening.

### 5.2 Data and modeling strategy

The data stemmed from a large registry where information collected at health screenings were linked to hospital discharge diagnoses and death certificates.^24^, ^25^ Approximately 13% of the relevant Austrian population participates in this health screening program every year, as it is offered free‐of‐charge. For the model development, a dataset with observations from 4769 men was available. The outcome variable of interest was time from the first health screening examination to occurrence of a fatal or nonfatal cardiovascular event during the study period, and the median follow‐up time was 3.9 years (interquartile range 2.6‐5.0 years). Such a cardiovascular event was observed in 4.86% (n = 232) participants. At the health screening, various characteristics were electronically recorded which could be potentially associated with the risk of cardiovascular disease since this screening program aims at preventing cardiovascular and other diseases. Based on discussions with internal medicine specialists and use in previously published cardiovascular risk prediction models,^26^, ^27^, ^28^ we preselected the following variables for our global model: age, systolic blood pressure, use of blood pressure lowering medication, cholesterol ratio, smoking status, diabetes, BMI score, physical activity (three categories), waist circumference (two categories), triglycerides, presence of protein in urine, and presence of glucose in urine. Their correlation structure is shown in Supplementary Material S10. In making this preselection, we assumed that all of these variables are associated with the outcome in a multivariable model, but for some of them the association might be negligible such that they could be omitted in a concise descriptive model. We estimated a global model with all preselected covariates in which approximately 18 events were available per variable (232 events/13 variables). Subsequently, we applied BE(AIC) using the R package *survival* to derive the more concise “selected” model.

The resulting estimates and stability measures are given in Table 4. To calculate stability measures, we used the subsampling approach [*m* = ⌊0.5*N* ⌋] to estimate VIF and MSF, and the bootstrap to estimate RMSDR and RCB. In both approaches, 1000 resamples were drawn. In addition, we calculated the PSFs and their SEs for the BE(AIC) model using the R package *shrink*.^29^

**TABLE 4:** Cardiovascular risk study: Global model, model selected with BE(AIC), stability measures, and parameterwise shrinkage factors

	Estimate, global	SE, global	VIF (%)	Estimate, selected	SE, selected	RMSDR	RCB (×100)	SF (1 + RCB)^−1^	SE, SF	PSF, selected	SE, PSF
Age in decades	0.718	0.070	100.0	0.709	0.069	0.944	0.850	0.992	0.090	1.002	0.097
Blood pressure lowering medication (yes vs no)	0.672	0.149	98.5	0.696	0.147	1.007	1.359	0.987	0.214	0.976	0.212
Smoking status (yes vs no)	0.660	0.157	98.2	0.685	0.152	1.078	1.222	0.988	0.236	0.927	0.223
Protein in urine (yes vs no)	0.558	0.212	68.2	0.551	0.211	1.343	9.984	0.909	0.250	0.849	0.391
Systolic blood pressure per 10 mmHg	0.077	0.038	54.3	0.082	0.037	1.355	25.434	0.797	0.235	0.728	0.452
Waist circumference (too large vs normal)	−0.311	0.156	46.4	−0.275	0.140	1.274	17.616	0.850	0.275	0.636	0.504
Diabetes (yes vs no)	0.326	0.201	39.0	0.374	0.192	1.271	44.691	0.691	0.194	0.745	0.522
Triglycerides per 10 mg/dL	0.012	0.008	32.3			1.225	39.079	0.719	0.218		
Ln(Total cholesterol/HDL cholesterol)	−0.213	0.248	13.3			1.080	109.393	0.478	0.293		
BMI score per 5 kg/m^2^	0.017	0.079	11.3			0.954	37.182	0.729	4.867		
Glucose in urine (yes vs no)	−0.185	0.399	6.7			1.612	333.120	0.231	0.372		
Physical activity sometimes vs never	−0.048	0.213	3.4			0.744	190.973	0.344	0.878		
regularly vs never	−0.067	0.218	3.4			0.789	219.165	0.313	0.525		

Abbreviations: BMI, body mass index; HDL, high‐density lipoprotein; PSF, parameterwise shrinkage factor; RCB, relative conditional bias; RMSDR, root mean squared difference ratio; SF, shrinkage factor; VIF, variable inclusion frequency.

### 5.3 Results

In Table 4, we ranked the variables by their VIFs. The most important predictors were age, smoking status and BP medication with VIFs of approximately 100%.

The MSF of the selected model was estimated as 6.0%. No other combination of variables yielded a higher MSF. Given the large number of different sets of covariates that would be possible to select, the value of the estimated MSF indicates relatively stable selection.

RMSDR was greater than one for most of the selected variables indicating that application of variable selection increased the variability of the estimated regression coefficients. This is in line with theoretical results see, for example, Hjort and Claeskens.^30^ Presence of protein in urine and systolic BP had RMSDR > 1.3, whereas the selection procedure decreased its mean squared error of physical activity by not selecting it.

RCB was positive for all variables. The positive bias in the selected model was negligible for strong variables selected in almost all resamples, whereas the RCB increased for variables that were less important and selected less often. Especially the regression coefficients of cholesterol ratio, glucose in urine and physical activity were highly biased if these variables were selected. Occasionally, the bootstrap‐based RCB can be positive even if the selected estimate is smaller in magnitude than the global estimate, which in our dataset occurs for the variable age.

We also computed the novel version of the SFs based on RCBs (see section 3.1), and estimated corresponding SEs via the delta method. Such SF can be read as an indicator of how much a regression coefficient has been overestimated. In the example, for the most important (strongest) variables (VIF ∼ 100%) it was close to 1 (no overestimation), while it was smaller for the other variables. Compared with PSFs^19^, ^29^, ^31^ the novel SFs have similar values with smaller SEs (in particular for lower numbers). Hence, just like PSFs, the novel SFs might also be useful to express the relative importance of each variable.

In this example, BE(AIC) suggested a reasonable, sparse descriptive model. No strong bias (RCB) was induced by variable selection and the SF is close to one for most of the selected covariates. Diabetes and triglycerides are two covariates that cannot be clearly identified as predictive or nonpredictive. Both have a relatively high RCB und a lower SF around 0.7. However, both covariates are competing for selection (pairwise inclusion frequency 5.5%; Supplementary Material 10) and the model selected diabetes but did not include triglycerides; without the additional information on model stability a researcher might easily overlook this arbitrary preference of the selection procedure for diabetes.

## 6 DISCUSSION

### 6.1 Optimal resampling approaches for calculating stability measures and beyond

Here, we first defined estimands for the four measures quantifying model stability that could be approximated using simulation, and investigated how accurately these estimands could be estimated by various resampling techniques. Subsampling seems favorable to estimate MSF and VIF, while estimation of RCB and RMSDR should better rely on bootstrapping. The choice for a suitable proportion of the original sample to be used for subsampling is delicate.^32^ Analytically, one can show that for a fixed model, bootstrapping generates approximately the same sampling distribution as subsampling one half of the original sample size (see Supplementary Material S1). Empirically, we found that this strategy works well in terms of accuracy of MSFs, and also of VIFs for nonpredictors, but higher subsampling proportions may yield higher accuracy to estimate VIFs of predictors.

While VIF, MSF, and RCB seem to be reliably estimated by resampling techniques, it is harder to quantify the effect of variable selection on the variation of the regression coefficients. In our simulation study, we found that RMSDR may be well approximated by the bootstrap only for predictors that are uncorrelated to other candidate covariates, a situation rarely encountered in practice. For other covariates, such as correlated predictors, or nonpredictors, the RMSDR is most often overestimated. Most likely this is a consequence of substituting the true model in the definition of the estimand by the global model when estimating RMSDR. We have also tried other resampling approaches in our analyses, such as the parametric or model‐based bootstrap, that is, drawing observations of the outcome variable given the estimated coefficients of regression model and adding the resampled residuals.^13^ The parametric bootstrap could start with the selected model, the global model or even mixing results over a set of different models such as those selected in a (nonparametric) bootstrap. In all these attempts, we found that accuracy was not improved and that, occasionally, underestimation of the RMSDR resulted. Because possible problems of variable selection procedures should not be underestimated, we have a preference for the nonparametric bootstrap to estimate RMSDR.

Moreover, we established a similarity between RCB and previously proposed PSFs.^19^ In our real data example our proposal of bias correcting SFs was more efficient (less variability) than PSFs. However, we have not further explored this possible efficiency gain, and one should keep in mind that the two proposals quantify the bias relative to different quantities (see section 3.1).

### 6.2 Expected behavior in more extreme situations

In our simulation study we defined marginal scenarios based on common recommendations on multivariable modeling.^3^, ^11^, ^33^ Specifically, we defined the most extreme scenario with 150 observations and 17 variables. In such situations one would already expect some overfit in the global model, which will further increase if the number of variables increases or if the number of observations decreases. For VIFs, Figure 1 suggests that precision of their estimates does not depend on sample size, but rather on a variable being a predictor or not, and on the absolute value of the estimate. Similar considerations apply to MSF (Table 3). Hence, VIF and MSF should also be suitable to indicate model instabilities in more extreme scenarios than those studied here.

However, we observed that precise estimation of RCB and in particular of RMSDR becomes difficult already in our marginal scenario (Supplemental Figures 5 and 6). When considering fewer observations or more variables, we must expect that median bias and median absolute bias will further increase and then in particular RMSDR must be even more cautiously interpreted. If an analyst is already alerted by low VIFs, RMSDR will not contribute to further characterize model instabilities.

### 6.3 Awareness of model instability and reporting of stability measures are essential

Software packages for regression modeling offer model selection but no standard software routinely reports any measures to quantify model instability. Such measures may confirm the stability of a model, may point at instabilities and may even indicate that the instabilities induced by a particular variable selection procedure are too severe to be compensated by the benefits of reporting the parsimonious model. In such a case, the modeling strategy could be modified by choosing a different, probably more conservative variable selection procedure or by more efficient use of background knowledge in the preselection of variables. If our measures were routinely reported in software for variable selection, data analysts would be immediately informed about possible instability problems which would otherwise often go unnoticed.

Our recommendations on quantities to report along with the results of variable selection procedure are summarized in Table 5 and implemented in the R package *abe*.^34^ This package provides the possibility to calculate the four stability measures by the simple bootstrap, by the m‐out‐of‐n bootstrap or by subsampling. According to our results and recommendations, the user could choose a sample size of *m* = 0.5*N* for subsampling to estimate the VIF for each variable considered for selection and the MSF of the most likely models. Likewise, the bootstrap could be employed to estimate RCB and RMSDR for each variable of the global model.

**TABLE 5:** Summary and recommendations for stability measures

Stability measure	What it should estimate (interpretation of the estimand)	How it should be estimated (resampling approach)	Rationale and *limitation*
Variable inclusion frequency	Probability of a variable to be selected in the final model	Subsampling with *m* = 0.5*N* (S_0.5_)	Consistent and efficient estimation for nonpredictor
Model selection frequency	Probability of a specific combination of variables to constitute the final model	Subsampling with *m* = 0.5*N* (S_0.5_)	Consistent and efficient estimation
Relative conditional bias	Bias of a selected regression coefficient relative to the true regression coefficient	Bootstrap	Consistent and efficient estimation
Root mean squared difference ratio	Inflation of the SE of a regression coefficient induced by the variable selection procedure	Bootstrap	Consistent for uncorrelated predictors *Overestimated for correlated predictors and for nonpredictors*

## Supporting information


**Appendix S1**: Supporting Information

## Data Availability

The original data used in the example section are not publicly available due to privacy and ethical restrictions. However, a synthetic data set is provided as part of the supplementary material to reproduce results. Simulated data can be reproduced from the supplementary material.
