# Supplementary material for: Selection of variables for multivariable models: Opportunities and limitations in quantifying model stability by resampling
Source: Stat Med. 2020 Oct 21;40(2):369–81. doi: 10.1002/sim.8779 (PMC7820988; doi:10.1002/sim.8779)
Supplement: Supplementary file 1 — Appendix S1: Supporting Information [file SIM-40-369-s001.pdf]

Supplementary Material

Wallisch C, Dunkler D, Rauch G, de Bin R, Heinze G

Selection of variables for multivariable models: opportunities and limitations in quantifying model stability by resampling, 2020.

Content

Supplementary Material S1: Equivalence of subsampling with $m = 0.5N$ and the bootstrap to obtain the sampling distribution of a regression coefficient 2

Supplementary Material S2: Distribution and correlation structure of design variables $xj, j = 1, \ldots, 17$. 3

Supplementary Material S3: Simulation study: Monte Carlo errors 5

Supplementary Material S4: Simulation study: Variable inclusion frequencies (VIF) for BE(AIC), BE(0.05) and Lasso 6

Supplementary Material S5: Simulation study: Model selection frequency for the correct model for BE(AIC), BE(0.05) and Lasso 8

Supplementary Material S6: Simulation study: Relative conditional bias (RCB) 9

Supplementary Material S7: Simulation study: Root mean squared difference (RMSD) ratio 10

Supplementary Material S8: Simulation study: Results for BE(AIC) in logistic regression 11

Supplementary Material S9: Simulation study: Results for BE(AIC) in Cox regression 13

Supplementary Material S10: Example: Correlation structure of variables and pairwise inclusion frequencies 15

References 17

Supplementary Material S1: Equivalence of subsampling with $m = 0.5N$ and the bootstrap to obtain the sampling distribution of a regression coefficient

Consider $\hat{\beta}$ the vector of estimates of regression coefficients from analysis of the original sample of size N. Let $\hat{\beta}^{(b)}$ be the vector of estimates of regression coefficients from analysis of the bth resample. If the nonparametric bootstrap (resampling N observations with resampling) was applied, the following is an estimate of the variance of $\hat{\beta}_j$, the jth regression coefficient:[1]

$$\frac{1}{B}\sum_{b=1}^{B}\left(\hat{\beta}_j^{(b)} - \hat{\beta}_j\right)^2$$

If the jackknife is used (resampling N data sets each consisting of $N - 1$ observations, resampling without replacement), then the variance estimator is given by

$$\frac{N-1}{N}\sum_{i=1}^{N}\left(\hat{\beta}_j^{(i)} - \hat{\beta}_j\right)^2$$

Here $\hat{\beta}_j^{(i)}$ is the regression coefficient of interest from the ith subsample, i.e., from the subsample where observation i has been left out. The formula can be rewritten to

$$\frac{N-1}{1}\cdot\frac{1}{N}\sum_{i=1}^{N}\left(\hat{\beta}_j^{(i)} - \hat{\beta}_j\right)^2$$

where $\frac{1}{N}\sum_{i=1}^{N}\left(\hat{\beta}_j^{(i)} - \hat{\beta}_j\right)^2$ denotes the expected squared deviation of a resampled regression coefficient from its original-sample counterpart. Note that the multiplicator $A/L = (N-1)/1$ is the ratio of included observations per resample divided by left-out observations per resample.

Now consider a general subsampling strategy of m observations without replacement. The expected value of leaving out $N - m$ observations is given by $\frac{1}{B}\sum_{b=1}^{B}\left(\hat{\beta}_j^{(b)} - \hat{\beta}_j\right)^2$, where $B = \binom{N}{m}$ could denote the number of different ways to draw m observations out of N. (One can approximate the full set of possible resamples by a random sample of, say, $B = 1000$ resamples.) Considering that now $A/L = m/(N-m)$, the variance of $\hat{\beta}_j$ with this subsampling scheme is well approximated by

$$\frac{m}{N-m}\cdot\frac{1}{B}\sum_{b=1}^{B}\left(\hat{\beta}_j^{(b)} - \hat{\beta}_j\right)^2$$

Thus, with $m = N - m$, or $m = 0.5N$, the distribution of the subsampled regression coefficient estimates the sampling distribution of the regression coefficient, just like the distribution of the bootstrapped regression coefficients.

Supplementary Material S2: Distribution and correlation structure of design variables $x_j, j = 1, \ldots, 17$

Supplementary Figure 1: Histograms of simulated design covariates $X_1, \ldots, X_{17}$ from 1,000 simulated observations.

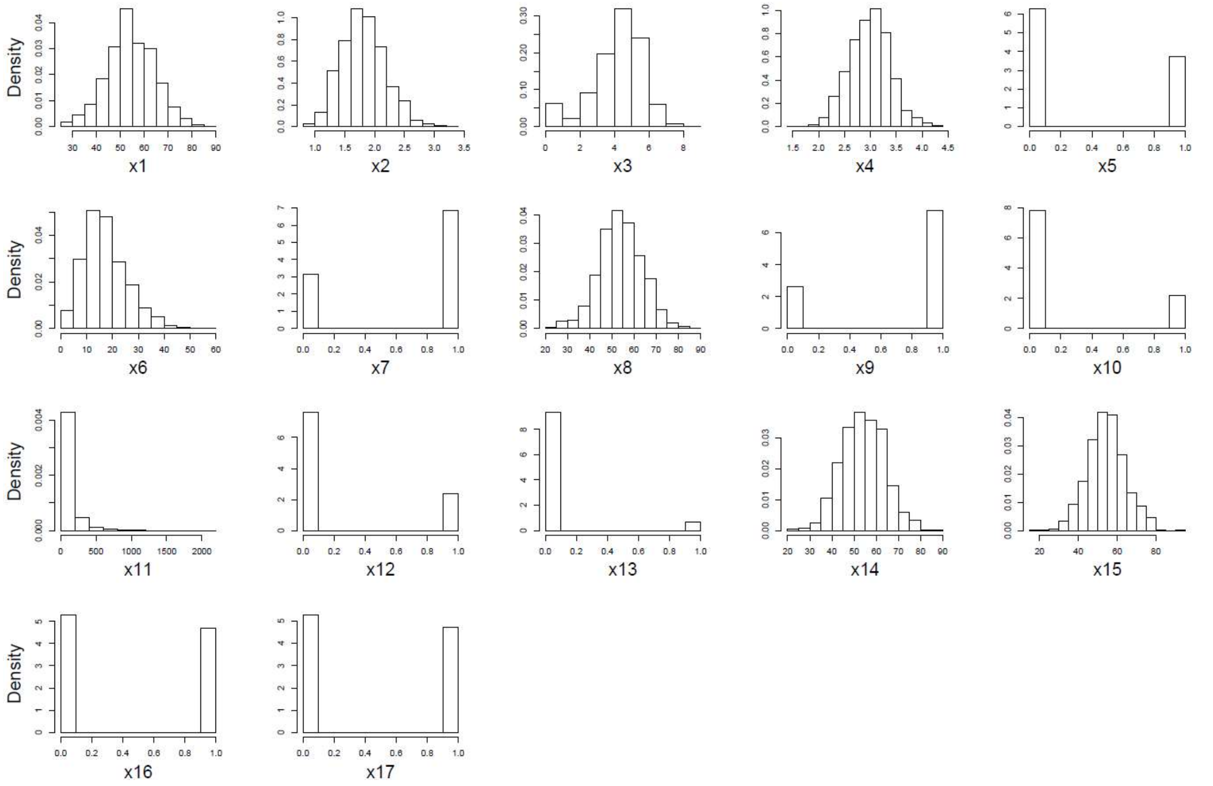

Supplementary Figure 2: Correlation network graph of all continuous (circle) and binary (square) design covariates. Numbers printed close to the edges are empirical correlation coefficients observed in a simulation of 1,000 observations. Edges are shown for a pair of covariates if the absolute value of their correlation coefficient exceeded 0.10. Widths of edges are proportional to correlation.

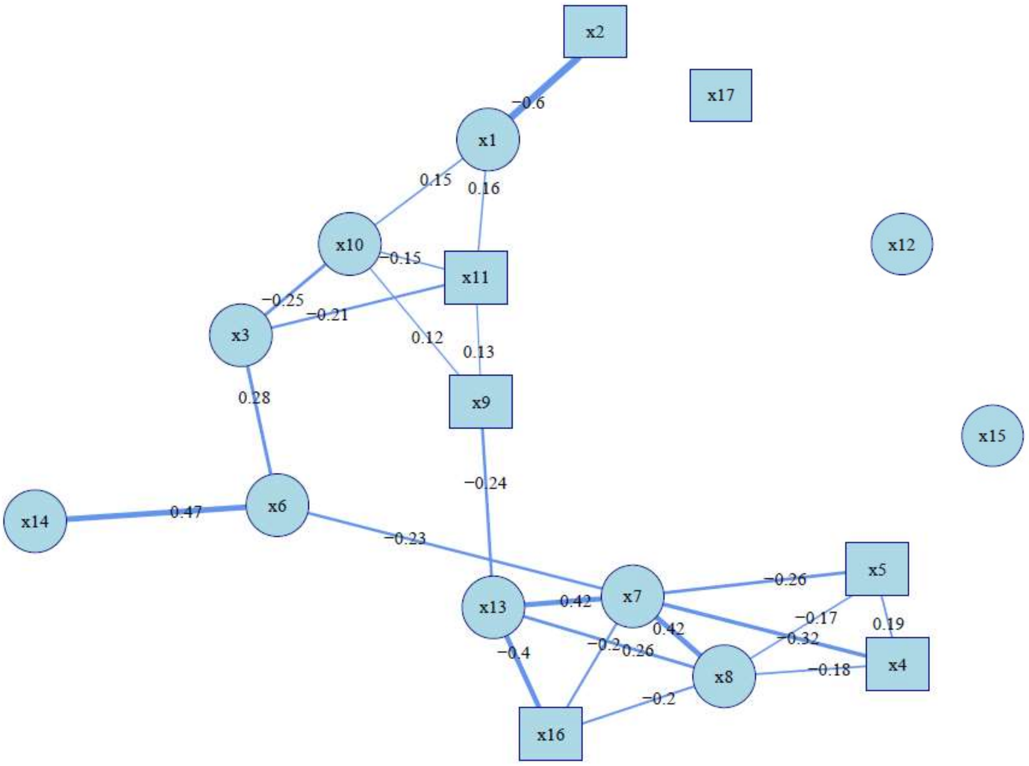

Supplementary Material S3: Simulation study: Monte Carlo errors

At a sample size of N=150, the Monte Carlo errors of the mean estimated MSF (x100) and of the RMSE of the estimated MSF (x100) were 0.01 in the bootstrap and in any subsampling approach. The following tables show the MCE of summary statistics of all other stability measures.

Supplementary Table 1: Monte Carlo error of summary statistics of stability measures with various resampling approaches, evaluated for all design covariates at a sample size of N=150.

Subsampling with $m = 0.5N$

	X1	X2	X3	X4	X5	X6	X7	X8	X9	X10	X11	X12	X13	X14	X15	X16	X17
mean estimated VIF (x100)	0.48	0.52	0.56	0.73	0.79	0.82	0.75	0.61	0.46	0.48	0.48	0.49	0.49	0.47	0.47	0.46	0.43
median estimated RCB (x100)	0.98	0.67	0.61	1.98	0.04	0.75	0.43	2.89	1.39	0.48	7.99	0.61	0.27	5.89	0.11	0.32	5.06
median estimated RMSDR	0.01	0.02	<0.01	<0.01	0.01	<0.01	<0.01	<0.01	0.02	<0.01	0.02	0.01	0.01	0.01	0.02	0.02	0.01
RMSE of estimated VIF (x100)	<0.01	<0.01	0.02	<0.01	0.02	0.03	<0.01	<0.01	0.02	<0.01	<0.01	<0.01	0.02	<0.01	<0.01	<0.01	<0.01
MAD of estimated RCB (x100)	0.96	0.12	<0.01	0.60	0.93	0.06	0.08	2.53									
MAD of estimated RMSDR	0.01	0.02	<0.01	0.01	0.02	<0.01	0.01	0.01	<0.01	<0.01	0.02	0.01	0.01	0.01	<0.01	0.02	0.01

Subsampling with $m = 0.632N$

	X1	X2	X3	X4	X5	X6	X7	X8	X9	X10	X11	X12	X13	X14	X15	X16	X17
mean estimated VIF (x100)	0.41	0.50	0.57	0.75	0.89	0.94	0.89	0.81	0.62	0.64	0.61	0.61	0.64	0.60	0.60	0.58	0.59
median estimated RCB (x100)	0.20	0.18	0.64	0.82	0.52	0.99	1.66	2.30	3.64	6.18	0.77	0.26	1.93	0.76	21.40	6.06	3.75
median estimated RMSDR	0.01	<0.01	<0.01	<0.01	0.02	<0.01	0.01	<0.01	0.03	0.01	0.01	0.01	<0.01	0.01	0.01	0.04	0.04
RMSE of estimated VIF (x100)	<0.01	<0.01	<0.01	0.02	0.02	0.03	0.02	<0.01	0.02	<0.01	<0.01	<0.01	<0.01	<0.01	0.02	<0.01	<0.01
MAD of estimated RCB (x100)	0.43	0.08	0.91	0.06	0.69	0.30	1.74	2.52									
MAD of estimated RMSDR	<0.01	<0.01	0.01	<0.01	<0.01	0.01	0.01	0.01	0.01	<0.01	0.03	<0.01	<0.01	0.01	0.01	0.01	<0.01

Subsampling with $m = 0.8N$

	X1	X2	X3	X4	X5	X6	X7	X8	X9	X10	X11	X12	X13	X14	X15	X16	X17
mean estimated VIF (x100)	0.29	0.46	0.53	0.80	1.03	1.05	1.08	1.07	0.85	0.90	0.83	0.83	0.83	0.84	0.81	0.79	0.79
median estimated RCB (x100)	0.19	0.09	0.69	0.03	0.29	0.78	3.35	0.21	4.51	9.97	0.80	6.45	10.46	0.52	4.59	0.40	0.51
median estimated RMSDR	0.01	0.02	0.01	<0.01	0.02	0.04	<0.01	<0.01	0.01	<0.01	0.05	0.01	<0.01	0.01	0.04	0.01	0.01
RMSE of estimated VIF (x100)	<0.01	<0.01	<0.01	<0.01	<0.01	<0.01	0.06	<0.01	<0.01	<0.01	<0.01	0.03	<0.01	<0.01	<0.01	<0.01	<0.01
MAD of estimated RCB (x100)	0.13	0.74	0.44	0.67	0.18	0.04	2.78	4.64									
MAD of estimated RMSDR	<0.01	<0.01	0.01	<0.01	0.02	0.04	0.01	0.02	0.03	<0.01	<0.01	<0.01	0.01	<0.01	0.01	<0.01	<0.01

Bootstrap

	X1	X2	X3	X4	X5	X6	X7	X8	X9	X10	X11	X12	X13	X14	X15	X16	X17
mean estimated VIF (x100)	0.33	0.39	0.47	0.60	0.71	0.73	0.73	0.68	0.55	0.53	0.59	0.54	0.57	0.57	0.56	0.56	0.56
median estimated RCB (x100)	0.07	0.31	0.50	0.27	0.93	0.39	0.69	5.96	1.71	0.19	1.27	5.19	0.94	0.46	8.07	10.25	0.80
median estimated RMSDR	<0.01	0.01	<0.01	<0.01	0.01	0.01	<0.01	0.01	0.01	0.01	0.03	0.01	0.01	0.01	0.01	0.01	<0.01
RMSE of estimated VIF (x100)	<0.01	<0.01	<0.01	0.02	0.02	0.03	0.02	<0.01	0.02	<0.01	<0.01	<0.01	<0.01	<0.01	0.02	<0.01	<0.01
MAD of estimated RCB (x100)	0.08	0.29	0.03	0.05	0.07	0.15	0.12	1.33									
MAD of estimated RMSDR	<0.01	<0.01	<0.01	0.01	<0.01	0.01	<0.01	<0.01	0.01	0.01	0.01	0.01	<0.01	0.01	0.01	0.01	<0.01

Supplementary Material S4: Simulation study: Variable inclusion frequencies (VIF) for BE(AIC), BE(0.05) and Lasso

Supplementary Figure 3: Mean estimated VIF by subsampling with $m = 0.5N$ (‘5’, blue), $m = 0.632N$ (‘6’, purple), $m = 0.8N$ (‘8’, red), by bootstrap (‘B’, yellow), and their estimands (‘X’, black). Variables are ranked by partial R².

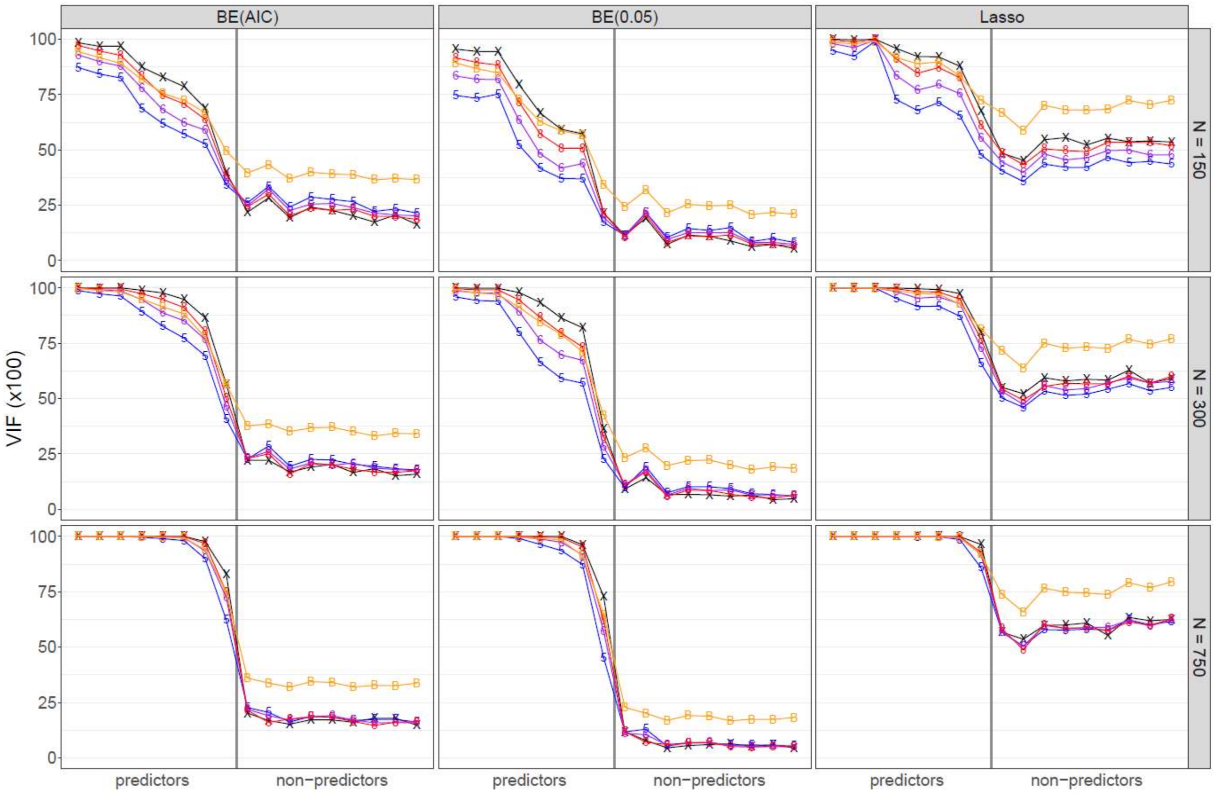

Supplementary Figure 4: Root mean squared error (RMSE) of estimated VIF by subsampling with $m = 0.5N$ ('5', blue), $m = 0.632N$ ('6', purple), $m = 0.8N$ ('8', red), by bootstrap ('B', yellow), and the omission/ selection strategy ('O', black). The omission/selection strategy sets the VIF estimate to 0 or 1 according to omission or selection in the model fitted on a simulated data set. Variables are ranked by partial R².

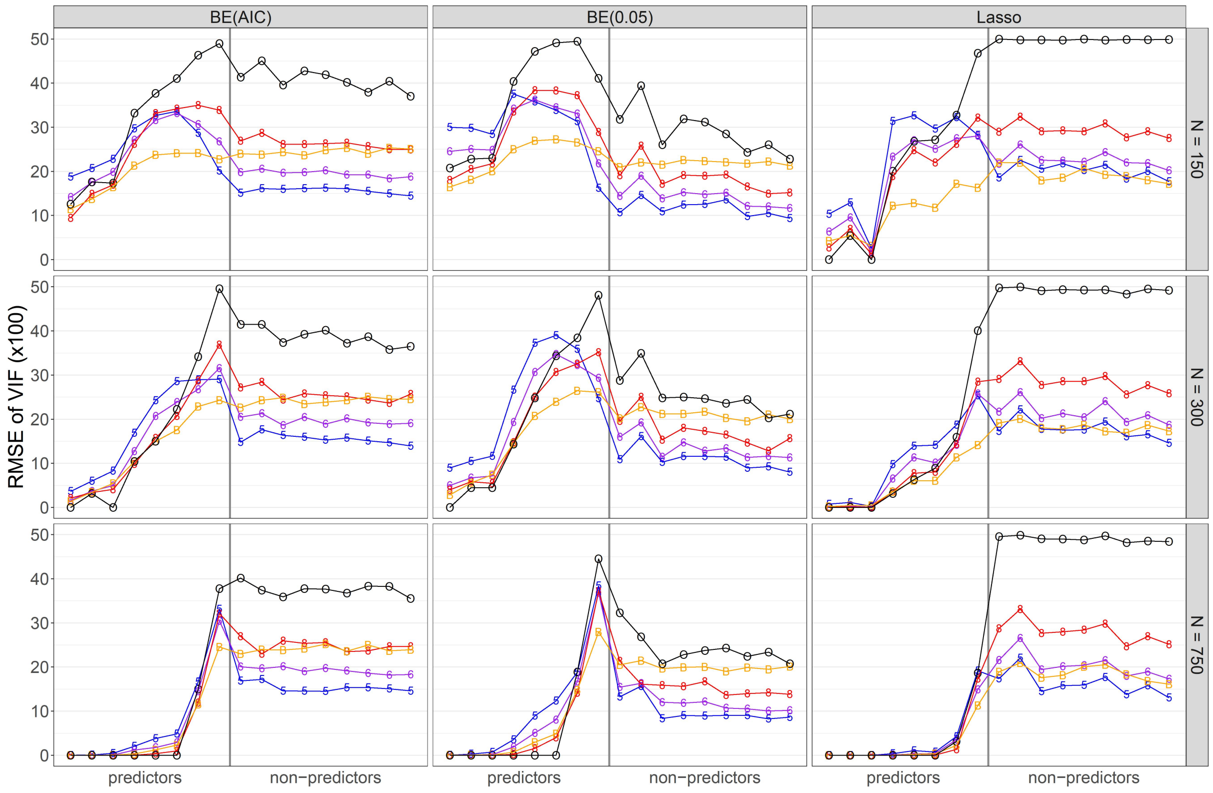

Supplementary Material S5: Simulation study: Model selection frequency for the correct model for BE(AIC), BE(0.05) and Lasso

Supplementary Table 2: Estimands, mean and RMSE of estimates of MSF for subsampling with $m = 0.5N$, $m = 0.632N$, and $m = 0.8N$ ($S_{0.5}$, $S_{0.632}$, $S_{0.8}$) and bootstrap (B) estimators. All numbers multiplied by 100.

BE(AIC)

	N=150		N=300		N=750	
Estimand	3.3		9.9		18.8	
Estimates	Mean	RMSE	Mean	RMSE	Mean	RMSE
$S_{0.5}$	0.4	2.8	2.9	7.9	12.4	12.1
$S_{0.632}$	1.0	3.2	5.1	8.4	14.8	13.9
$S_{0.8}$	1.5	5.0	7.3	13.5	16.8	20.7
B	0.3	3.6	1.0	8.9	2.4	16.3

BE(0.05)

	N=150		N=300		N=750	
Estimand	2.7		15.7		46.4	
Estimates	Mean	RMSE	Mean	RMSE	Mean	RMSE
$S_{0.5}$	0.1	2.0	2.3	13.6	22.7	28.5
$S_{0.632}$	0.4	3.2	5.4	14.0	32.2	27.4
$S_{0.8}$	1.0	4.6	9.8	17.7	37.5	31.6
B	0.6	3.2	3.4	13.4	12.1	36.8

Lasso

	N=150		N=300		N=750	
Estimand	0.1		0.3		0.3	
Estimates	Mean	RMSE	Mean	RMSE	Mean	RMSE
$S_{0.5}$	0.1	0.2	0.2	0.4	0.3	0.5
$S_{0.632}$	0.2	0.4	0.3	0.6	0.3	0.7
$S_{0.8}$	0.2	0.8	0.3	1.2	0.4	1.3
B	0.0	0.3	0.0	0.4	0.0	0.5

Supplementary Material S6: Simulation study: Relative conditional bias (RCB)

Supplementary Figure 5: Relative conditional bias. Upper rows: median estimate; middle rows: median deviation to the estimand; lower rows: median absolute deviation to the estimand; red and blue indicate high and low multiple correlation of a variable with others; solid and dashed lines represent predictors and non-predictors. The line width is proportional to absolute effect size.

A. RCB for BE(AIC) estimated by subsampling with $m = 0.5N$ ($S_{0.5}$), $m = 0.632N$ ($S_{0.632}$), $m = 0.8N$ ($S_{0.8}$), and by bootstrap

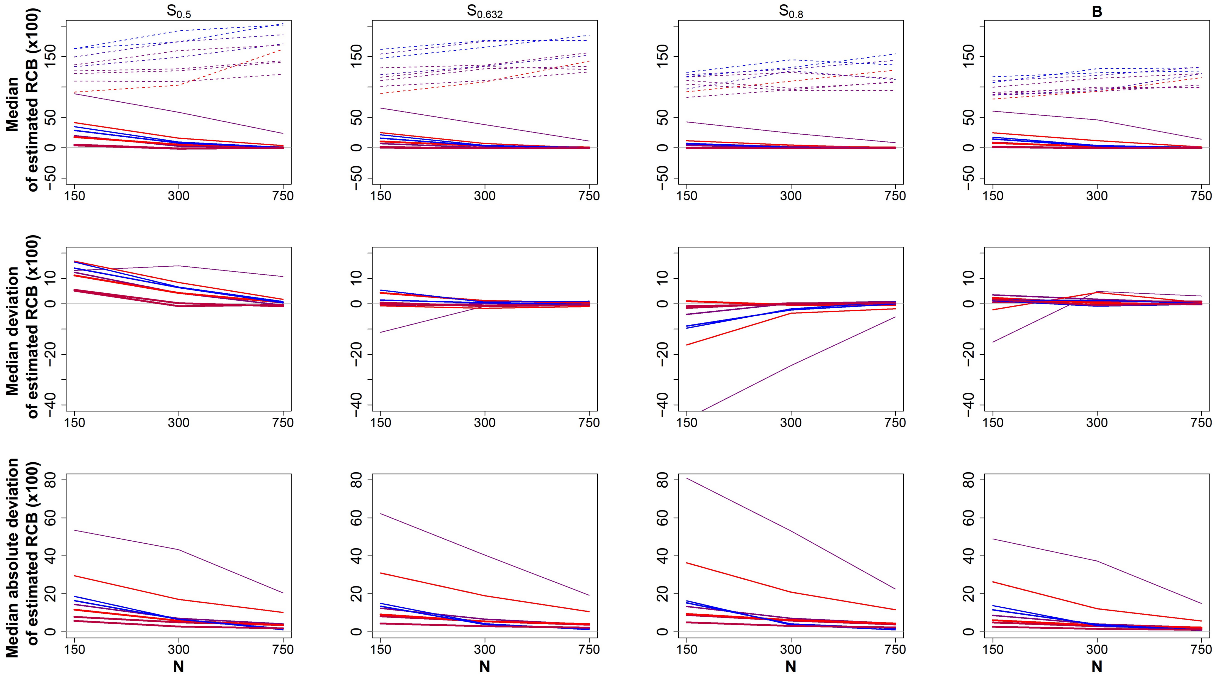

B. RCB estimated by bootstrap for BE(AIC), BE(0.05) and Lasso

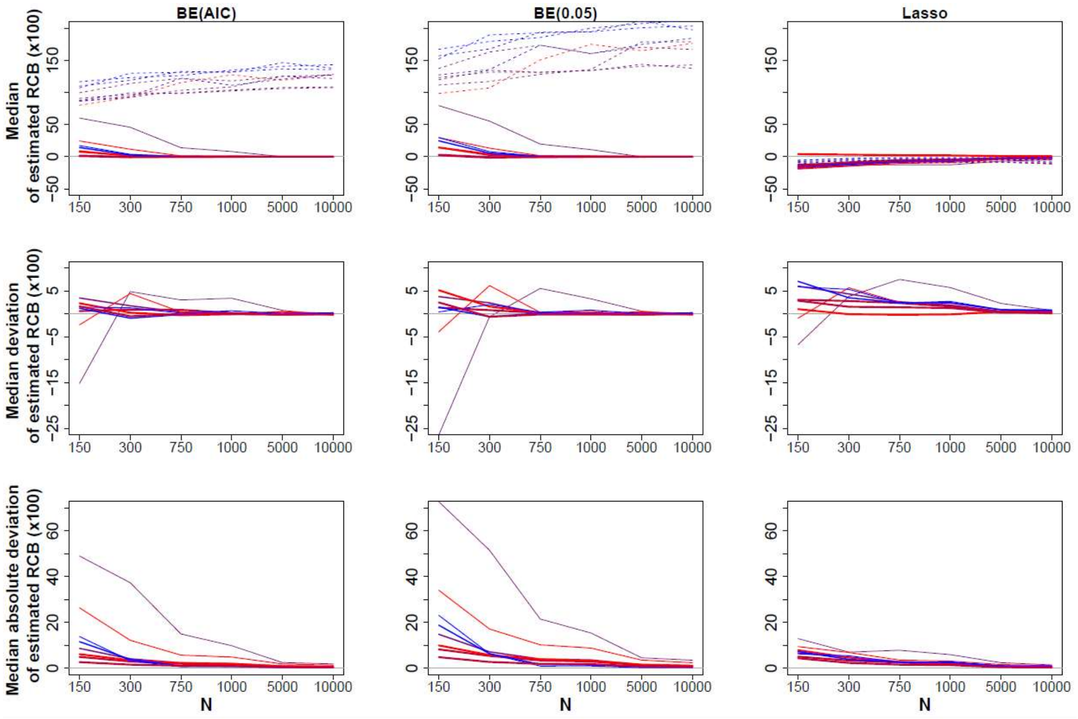

Supplementary Material S7: Simulation study: Root mean squared difference (RMSD) ratio

Supplementary Figure 6: Root mean squared difference ratio. Upper rows: median estimate; middle rows: median deviation to the estimand; lower rows: median absolute deviation to the estimand; red and blue indicate high and low multiple correlation of a variable with others; solid and dashed lines represent predictors and non-predictors. The line width is proportional to absolute effect size.

A. RMSD ratio for the global model estimated by subsampling with $m = 0.5N$ ($S_{0.5}$), $m = 0.632N$ ($S_{0.632}$), $m = 0.8N$ ($S_{0.8}$), and by bootstrap

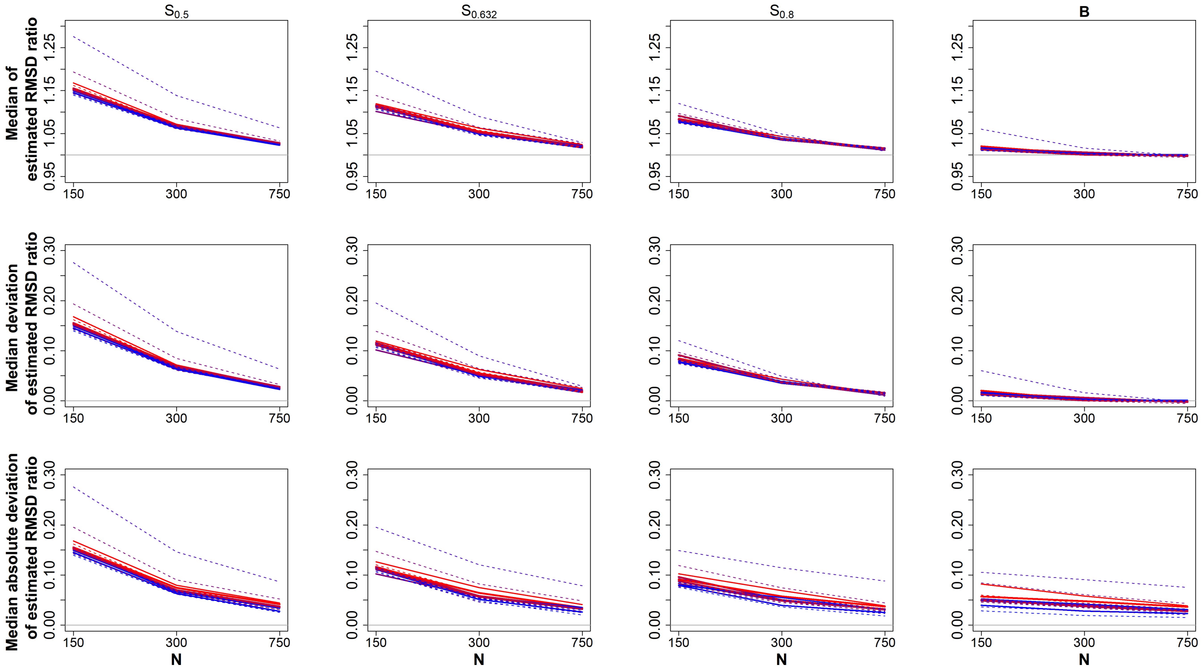

B. RMSD ratio estimated by bootstrap for BE(AIC), BE(0.05) and Lasso

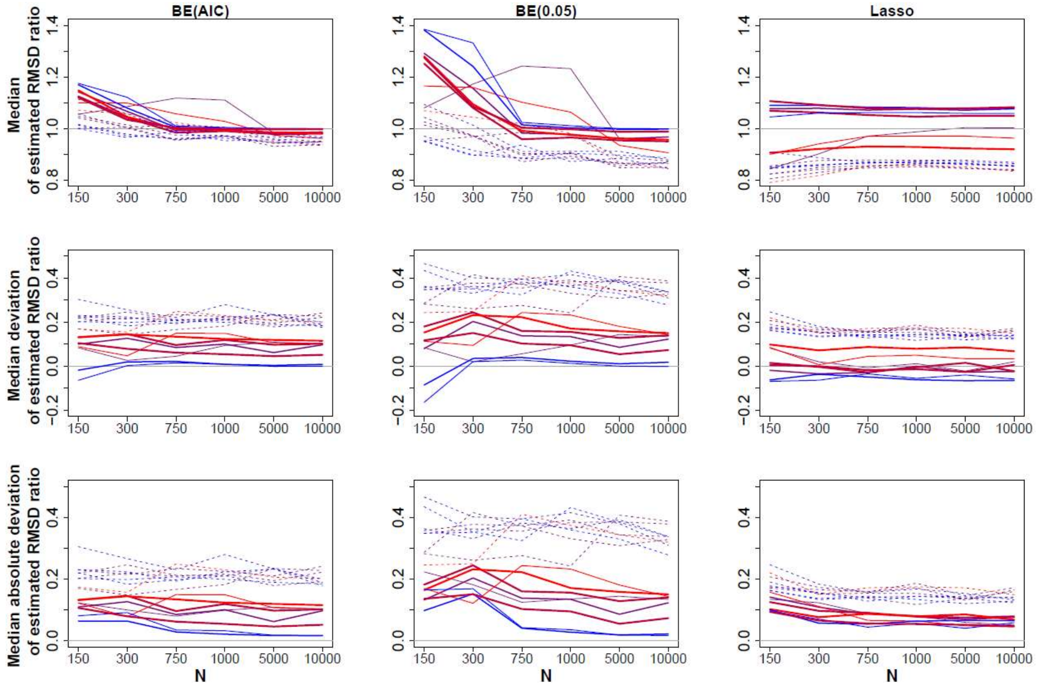

Supplementary Material S8: Simulation study: Results for BE(AIC) in logistic regression

Additional information to the simulation setup for logistic regression

The simulation setup for logistic regression was analogous to the setup for linear regression described in the manuscript up to the definition of the linear predictor η. The binary outcome Y_B was drawn from a Bernoulli distribution with event probability $\pi = [1 + \exp(-\eta + 8.47)]^{-1}$ which yielded a Nagelkerke's R^2 of 0.18 and an expected event rate of approximately 0.5.[2]

Supplementary Figure 7: Variable inclusion frequencies (VIF) in logistic regression

Left column: mean estimated VIF by subsampling with $m = 0.5N$ ('5', blue), $m = 0.632N$ ('6', purple), $m = 0.8N$ ('8', red), by bootstrap ('B', yellow), and their estimands ('X', black).

Right column: root mean squared error (RMSE) of estimated VIF by subsampling with $m = 0.5N$ ('5', blue), $m = 0.632N$ ('6', purple), $m = 0.8N$ ('8', red), by bootstrap ('B', yellow), and the omission/ selection strategy ('O', black). The omission/selection strategy sets the VIF estimate to 0 or 1 according to omission or selection in the model fitted on a simulated data set.

Variables are ranked by partial R^2.

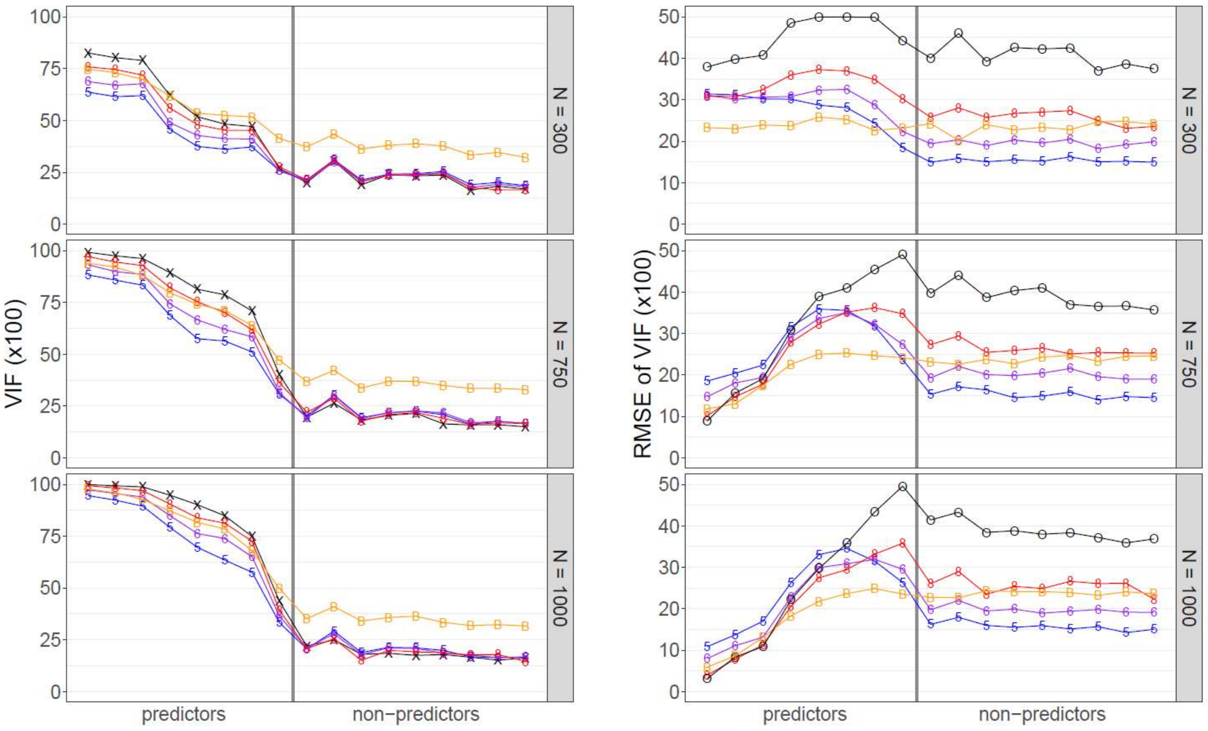

Supplementary Table 3: Model selection frequency for the correct model in logistic regression

Estimands, mean and RMSE of estimates for subsampling with $m = 0.5N$, $m = 0.632N$, and $m = 0.8N$ ($S_{0.5}$, $S_{0.632}$, $S_{0.8}$) and bootstrap (B) estimators. All numbers multiplied by 100.

	N=300		N=750		N=1000	
Estimand	0.2		3.5		5.7	
Estimates	Mean	RMSE	Mean	RMSE	Mean	RMSE
$S_{0.5}$	0.0	0.1	0.5	3.0	1.1	5.0
$S_{0.632}$	0.0	0.3	1.0	4.1	2.3	5.5
$S_{0.8}$	0.1	0.6	2.0	6.2	3.8	9.1
B	0.0	0.3	0.3	3.4	0.6	4.6

Supplementary Figure 8: Relative conditional bias (left column) and root mean squared difference ratio (right column) estimated by bootstrap in logistic regression
Upper row: median estimate; middle row: median deviation to the estimand; lower row: median absolute deviation to the estimand; red and blue indicate high and low multiple correlation of a variable with others; solid and dashed lines represent predictors and non-predictors. The line width is proportional to absolute effect size.

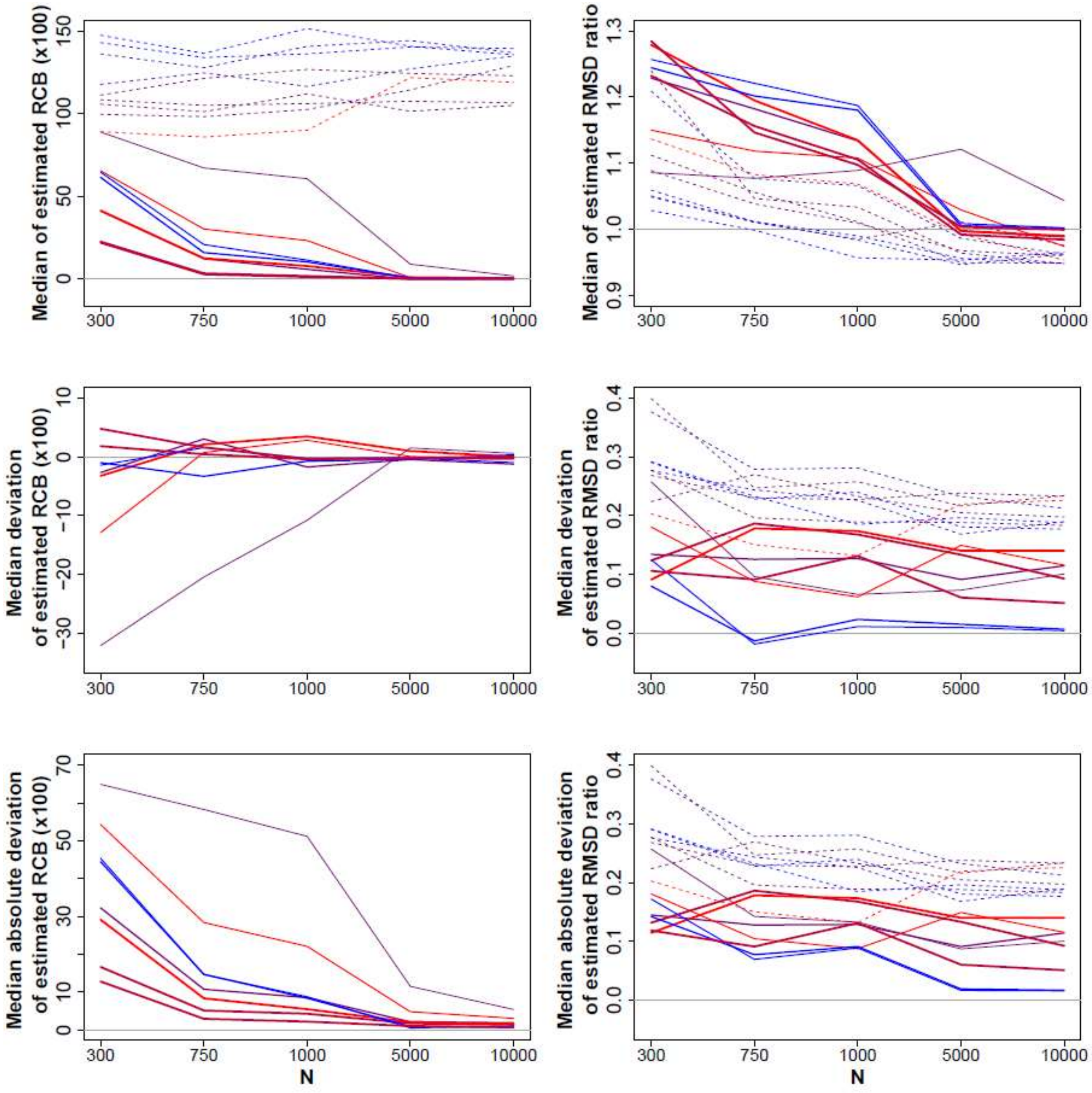

Supplementary Material S9: Simulation study: Results for BE(AIC) in Cox regression

Additional information to the simulation setup for Cox regression
The simulation setup for Cox regression was analogous to the setup for linear regression described in the manuscript up to the definition of the linear predictor η. To obtain a time-to-event outcome, Weibull distributed survival times T were drawn from $\left(-\log(U)/(\frac{1}{5}\exp(\eta - 3.5))\right)^{1/3}$,[3] whereby U was standard uniformly distributed. The follow-up times C were drawn from a uniform distribution $U(0.001, 3.851)$. The observable survival time was defined as $Y_T = \min(T, C)$ and the status indicator δ was 1 if $T < C$ and 0 otherwise. This setup yielded a censoring rate of approximately 0.5 and a Schemper-Henderson V of 0.22.[4]

Supplementary Figure 9: Variable inclusion frequencies (VIF) for Cox regression
Left column: mean estimated VIF by subsampling with $m = 0.5N$ ('5', blue), $m = 0.632N$ ('6', purple), $m = 0.8N$ ('8', red), by bootstrap ('B', yellow), and their estimands ('X', black).
Right column: root mean squared error (RMSE) of estimated VIF by subsampling with $m = 0.5N$ ('5', blue), $m = 0.632N$ ('6', purple), $m = 0.8N$ ('8', red), by bootstrap ('B', yellow), and the omission/ selection strategy ('O', black). The omission/selection strategy sets the VIF estimate to 0 or 1 according to omission or selection in the model fitted on a simulated data set.
Variables are ranked by partial R^2.

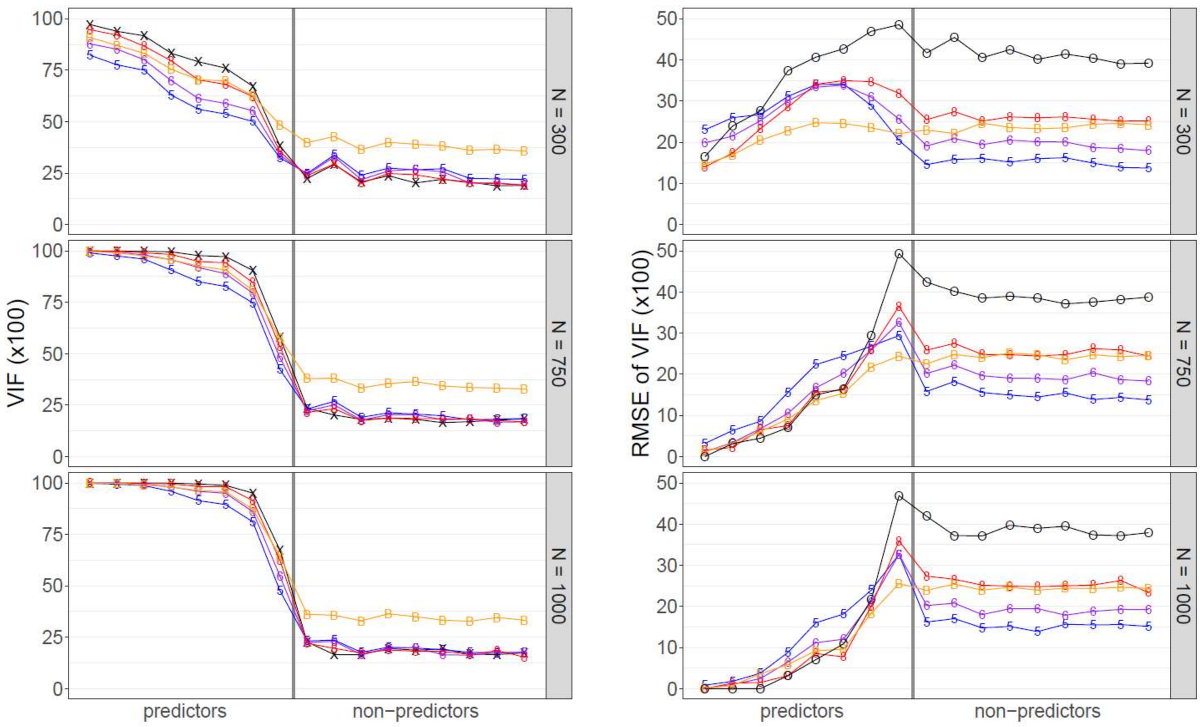

Supplementary Table 4: Model selection frequency for the correct model for Cox regression
Estimands, mean and RMSE of estimates for subsampling with $m = 0.5N$, $m = 0.632N$, and $m = 0.8N$ ($S_{0.5}$, $S_{0.632}$, $S_{0.8}$) and bootstrap (B) estimators. All numbers multiplied by 100.

	N=300		N=750		N=1000	
Estimand	2.6		10.4		13.3	
Estimates	Mean	RMSE	Mean	RMSE	Mean	RMSE
$S_{0.5}$	0.2	3.1	3.9	7.7	6.1	8.7
$S_{0.632}$	0.6	2.5	6.2	9.5	9.3	11.4
$S_{0.8}$	1.2	4.9	8.8	14.9	11.6	16.2
B	0.2	2.3	1.2	7.3	1.7	11.7

Supplementary Figure 10: Relative conditional bias (left column) and root mean squared difference ratio (right column) estimated by bootstrap for Cox regression
Upper row: median estimate; middle row: median deviation to the estimand; lower row: median absolute deviation to the estimand; red and blue indicate high and low multiple correlation of a variable with others; solid and dashed lines represent predictors and non-predictors. The line width is proportional to absolute effect size.

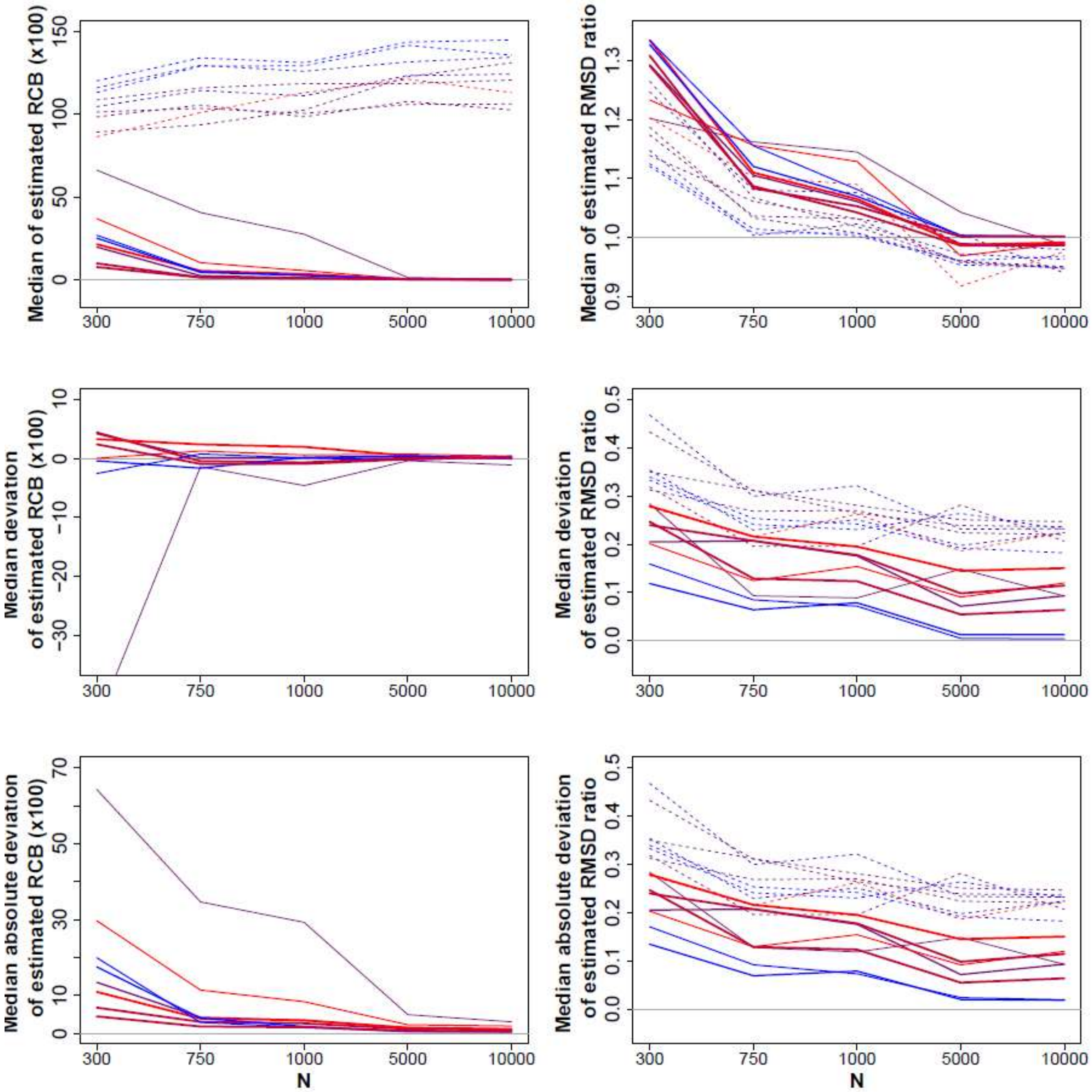

Supplementary Material S10: Example: Correlation structure of variables and pairwise inclusion frequencies

Supplementary Figure 11: Correlation network graph of all continuous (circle) and binary (square) covariates. Numbers printed close to the edges are empirical correlation coefficients. Edges are shown for a pair of covariates if the absolute value of their correlation coefficient exceeded 0.15. Widths of edges are proportional to correlation.

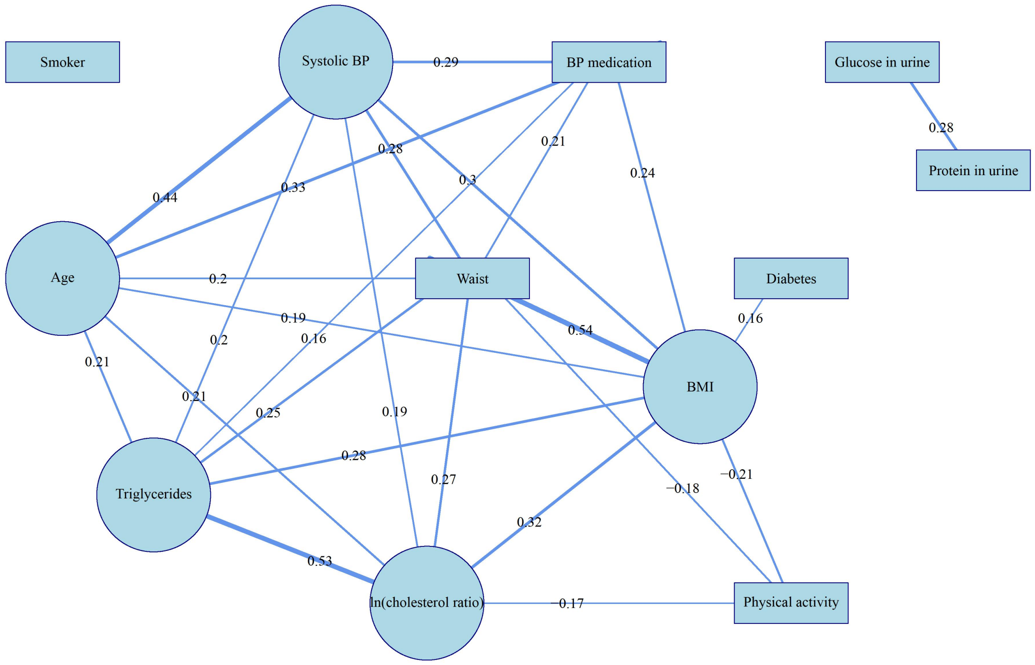

Supplementary Table 5: Pairwise inclusion frequencies of variables estimated by subsampling with $m = 0.5N$

"(+)" and "(-)" indicate if a pair of variables were selected significantly more or less often than by chance applying a significance level of 0.01. This was determined by conducting X^2-tests for independence.

	Age	BP lowering medication	Smoking status	Protein in urine	Systolic BP	Waist circumference	Diabetes	Tri-glycerides	Ln(Total chol/ HDL chol)	BMI score	Glucose in urin	Physical activity
Age	100	98.5	98.2	68.2	54.3	46.4	39	32.3	13.3	11.3	6.7	3.4
BP lowering medication		98.5	96.7	67.2	53.2	45.7	37.7 (-)	32.2	13.2	11.2	6.6	3.3
Smoking status			98.2	66.5	53.5	45.8	38.7	31.3	13	11.2	6.5	3.1
Protein in urine				68.2	36.3	34.4 (+)	25.1	22.9	9.4	6.7	4.3	2.2
Systolic BP					54.3	29.1 (+)	21.6	15.6	7.3	6.1	3.3	1.9
Waist cirumference						46.4	18.8	16.8	4.6 (-)	6.4	2.1	1.6
Diabetes							39.0	5.5 (-)	3.6	3.7	3.9 (+)	1.7
Triglycerides								32.3 (-)	9.6 (+)	3.9	1.6	1
Ln(Total chol/ HDL chol)									13.3	1.4	0.9	0.2
BMI score										11.3	0.7	0.2
Glucose in urine											6.7	0.5
Physical activity												3.4

BMI, body mass index; BP, blood pressure; chol, cholesterol; HDL, high-density lipoprotein

References

1. Efron B, Tibshirani RJ. *An introduction to the bootstrap.* London: Chapman & Hall; 1993.
2. Nagelkerke NJD. A note on a general definition of the coefficient of determination. *Biometrika.* 1991;78(3):691-692.
3. Bender R, Augustin T, Blettner M. Generating survival times to simulate Cox proportional hazards models. *Stat Med.* 2005;24(11):1713-1723.
4. Schemper M, Henderson R. Predictive accuracy and explained variation in Cox regression. *Biometrics.* 2000;56(1):249-255.
